# Clinical characteristics and risk factors of mirror syndrome: a retrospective case-control study

**DOI:** 10.1186/s12884-021-04143-3

**Published:** 2021-09-28

**Authors:** Zhenyan Han, Xiaodan Chen, Qingqing Wang, Jin Zhou, Yan Guo, Hongying Hou, Yuan Zhang

**Affiliations:** Department of Obstetrics and Gynecology, Third Affiliated Hospital of Sun Yat-sen University, No. 600 Tianhe Road, Tianhe District, Guangzhou, 510630 Guangdong Province China

**Keywords:** Mirror syndrome, Fetal hydrops, Placental thickening, Hemodilution

## Abstract

**Background:**

Mirror syndrome (MS) is a rare obstetric disorder complicated with high maternal morbidity and fetal mortality. MS is often misdiagnosed or underdiagnosed due to the low incidence and lack of awareness of its diverse features. This study aimed to summarise the etiology, clinical characteristics, and risk factors of MS among mothers with fetal hydrops.

**Methods:**

This retrospective case-control study included 37 pregnant women with fetal hydrops in the second and third trimesters from 58,428 deliveries performed at the Third Affiliated Hospital of Sun Yat-Sen University between January 2012 and December 2020. Cases were categorized as MS and non-MS according to the presence or absence of maternal mirroring symptoms. Binary logistic regression was performed for analysis.

**Results:**

Fourteen women developed MS with an overall incidence of 0.024% (14/58,428) and 37.8% (14/37) in the fetal hydrops cases. Among the 11 MS cases with known associated etiologies, seven had alpha thalassemia major. Onset of fetal hydrops was later (27.8 vs. 23.0 weeks) and the rate of placental thickening was higher (85.7% vs. 34.8%) in the MS group than in the non-MS group (*P* < 0.05). Regarding maternal characteristics, the MS group had higher maternal morbidity (85.7% vs. 8.7%), more weight gain (9.0 vs. 5.5 kg), higher rates of hypertension (35.7 vs. 0%) and proteinuria (64.3% vs. 4.3%), and lower levels of hemoglobin (88 vs. 105 g/L) and serum albumin (25.8 vs. 35.0 g/L) than the non-MS group (*P* < 0.05). Logistic regression analysis showed that onset of fetal hydrops at ≥24 weeks and placental thickening were associated with the risk of MS among fetal hydrops cases (OR 15.83, 95% CI 1.56–160.10 and OR 8.63, 95% CI 1.29–57.72, respectively).

**Conclusions:**

MS is relatively common among fetal hydrops cases in the late second and third trimesters, and alpha thalassemia major is the main etiology for fetal hydrops and also MS in this population. Complicated with high maternal morbidity, the key maternal features of MS include more weight gain, hemodilution, and hypertension. Among those with fetal hydrops, the onset time of ≥24 weeks and placental thickening are risk factors for MS.

**Supplementary Information:**

The online version contains supplementary material available at 10.1186/s12884-021-04143-3.

## Introduction

Mirror syndrome (MS) also known as “triple edema” or Ballantyne syndrome, is a rare disorder affecting pregnant women [1]. It describes the unusual association of fetal hydrops and placental edema with diverse maternal edematous manifestations [1–3]. As most of the published studies in the literature on MS consist of sporadic case reports that mainly focused on severe presentations, its incidence and pathophysiology are remain unclear [2, 3]. In fact, MS is often underdiagnosed or misdiagnosed due to unnoticed maternal symptoms of an abnormal fetus, or unawareness of MS and sometimes its preeclampsia-like manifestations [4]. Some researchers have hypothesized that MS shares a similar pathogenesis to preeclampsia with the same pattern of increased inflammatory markers or placental factors, which could be used as a diagnostic tool for MS; however, there is insufficient evidence to support this theory [5–8].

It has been reported that maternal morbidity usually increases in MS, and fetal mortality has been reported to be as high as 67.26% [3]. Most patients require induction of labor or termination of pregnancy because of irreversible fetal hydrops and poor prognosis. However, until now, the characteristics of MS have not been fully elucidated, and the risk factors for MS among hydropic fetuses are still unknown due to paucity of cases. In this study, we retrospectively compared MS cases with non-MS fetal hydrops controls to analyze the clinical characteristics and risk factors of MS.

## Methods

### Study design and subjects

A total of 59 pregnant women with fetal hydrops were retrospectively reviewed from 58, 428 deliveries at the Third Affiliated Hospital of Sun Yat-Sen University between January 2012 and December 2020. Twenty-two patients were excluded since they terminated their pregnancies in the first trimester and had no maternal complications. The remaining 37 patients who were in the second and third trimesters were divided into the MS and the non-MS groups according to the presence or absence of maternal mirroring symptoms. This study was approved by the Institutional Review Board of the Third Affiliated Hospital of Sun Yat-Sen University and written informed consent was obtained from each participant.

### Data collection

Maternal data included age, primiparity, body weight gain (kg), blood pressure (mmHg), hemoglobin (g/L), hematocrit (%), platelets (× 10^9^/L), albumin (g/L), uric acid (μmol/L), creatinine (μmol/L) and urinary protein. Fetal features included the time of onset of fetal hydrops (gestational weeks), site of edema, structural anomalies, volume of amniotic fluid, and placental thickness. Maternal and fetal outcomes included gestational weeks at time of delivery, mode of delivery, complications, transfusion of blood products, intensive care unit (ICU) admission, length and cost of hospital stay, neonatal death or survival, birth weight, placental weight, and pathologic examination.

### Diagnostic criteria

The diagnostic criteria were as follows: a) Fetal hydrops was defined as the pathological fluid accumulation in ≥2 different fetal compartments, including skin edema (skin thickness > 5 mm), ascites, pleural effusion, pericardial effusion, and other sonographic findings such as placental thickening or polyhydramnios (atypical fetal hydrops manifests as simple skin edema or body cavity effusion) [9]; b) placental thickening, indicated by ultrasound-detected placental thickness of ≥40 or ≥ 60 mm in the second and third trimesters, respectively [9]; c) placental edema confirmed by pathology of the placenta after delivery; and d) for MS, maternal edematous manifestations characterized by accumulation of excessive fluid within subcutaneous and body cavities, weight gain, and preeclampsia-like symptoms, which were associated with fetal hydrops and placental edema.

### Statistical analysis

Data are presented as median (interquartile range) for continuous variables and number (percentage) for categorical variables. Differences in continuous variables were compared between groups using Mann-Whitney U test. Fisher’s exact test was performed to analyze the categorical variables. The risk factors for MS were analyzed using binary logistic regression model. Data analysis was performed using SPSS statistical software version 27.0 (IBM; Armonk, NY, USA). A *P*-value of < 0.05 was considered statistically significant.

## Results

Among the 37 pregnant women with fetal hydrops, 14 (37.8%) patients who developed MS were enrolled in the MS group and the remaining 23 cases in the non-MS group. The overall incidence of MS in this population was 0.024% (14/58, 428). Genetic analysis for fetal hydrops was conducted in 27 cases, and 17 fetuses were diagnosed with alpha thalassemia major. The other known associated etiologies included twin-to-twin transfusion syndrome (TTTS) (3/37), structural cardiac abnormalities (2/37), congenital cystic adenomatoid malformation (1/37), fetal anemia (1/37), and placental chorioangioma (1/37). All 23 patients in the non-MS group and 12 patients in the MS group opted to terminate their pregnancies.

### Clinical features and outcomes of MS

Among the14 cases, the time of diagnosis of MS ranged from 22.6 to 34 gestational weeks. The etiology was identified in 11 cases and included alpha thalassemia major (7/14), structural cardiac abnormalities (2/14), TTTS (1/14), and fetal anemia (1/14). Prenatal sonographic findings indicated fetal hydrops (14/14), placental thickening (12/14), polyhydramnios (6/14), and oligohydramnios (3/14). Maternal clinical manifestations included edema (14/14: lower limb edema [10/14], ascites [6/14], pleural effusion, [5/14]), anemia (14/14), hypoalbuminemia (12/14), proteinuria (7/14) and hypertension (5/14). After delivery, placental edema was confirmed in all 14 cases by both gross and histologic placental examinations (Table S1).

Of the 12 pregnant women who opted for termination, seven patients had uneventful vaginal deliveries, and five underwent a cesarean section for various indications, which included maternal complications and failed labor induction. The other two patients for expectant management eventually underwent emergency cesarean section due to critical maternal conditions; two very preterm twin infants survived and one preterm neonate died. Maternal complications occurred in 12 cases, including postpartum hemorrhage (7/14), placenta accreta (5/14), acute left heart failure (3/14), renal dysfunction (3/14), pulmonary edema (2/14), HELLP (hemolysis, elevated liver enzymes, low platelet count) syndrome (2/14), liver dysfunction (1/14), placental abruption (1/14), and disseminated intravascular coagulation (DIC) (1/14). The ICU admission rate was 42.9% (6/14), and the median time for maternal symptoms to resolve was 7.5 days (range: 2–10 days) after delivery.

### Differences between MS and non-MS groups

Compared to the non-MS group, the median gestational age at fetal hydrops onset was significantly later (27.8 vs. 23.0 weeks, *P* = 0.003) and the proportion of placental thickening was higher (85.7% vs. 34.8%, *P* = 0.006) in the MS group. Regarding maternal symptoms, pregnant women in the MS group had more weight gain (9.0 vs. 5.5 kg, *P* = 0.003) and higher rates of hypertension (35.7% vs. 0%, *P* = 0.005), anemia (100% vs. 65.2%, *P* = 0.015), and proteinuria (64.3% vs. 4.3%, *P* < 0.001). Moreover, their hemoglobin levels (88 vs. 105 g/L, *P* < 0.001), platelet counts (155 vs. 237 × 10^9^/L, *P* = 0.005), and serum albumin levels (25.8 vs. 35.0 g/L, *P* < 0.001) were significantly lower. In contrast, the levels of uric acid (546 vs. 290 μmol/L, *P* = 0.042) and creatinine (59 vs. 44 μmol/L, *P* = 0.002) were higher in the MS group. As for outcomes, the rates of cesarean section, postpartum hemorrhage, blood product transfusion, and ICU admission were significantly higher in the MS group than in the non-MS group (50% vs. 0%, *P* < 0.001; 50% vs. 4.3%, *P* = 0.002; 64.3% vs. 4.3%, *P* < 0.001; and 42.9% vs. 0%, *P* = 0.001; respectively). Meanwhile, the median length of hospital stay was longer and the costs were significantly higher in the MS group (Table 1).

**Table 1:** Comparison of clinical data between mirror syndrome group and non-mirror syndrome group

Clinical Profiles	Non-mirror group (*n* = 23)	Mirror group (*n* = 14)	*P* value
**Fetal features**			
Time of fetal hydrops onset (wks)	23.0 (9.4)	27.8 (4.7)	0.003
Placental thickening	8/23 (34.8%)	12/14 (85.7%)	0.006
Polyhydramnios	4/23 (17.4%)	6/14 (42.9%)	0.132
**Maternal features**			
Maternal age (years)	28.0 (5.0)	28.5 (9.0)	0.699
Primipara	11/23 (47.8%)	4/14 (28.6%)	0.314
Body weight gain	5.5 (6.0)	9.0 (8.0)	0.003
Hypertension	0/23 (0)	5/14 (35.7%)	0.005
Systolic Blood Pressure (mmHg)	115 (12)	131 (24)	< 0.001
Diastolic Blood Pressure (mmHg)	64 (15)	82 (13)	< 0.001
Anemia	15/23 (65.2%)	14/14 (100%)	0.015
Hemoglobin (g/L)	105 (31)	88 (31)	< 0.001
Hematocrit (%)	31.8 (3.6)	26.4 (7.1)	< 0.001
Platelet counts (×10^9^/L)	237 (91)	155 (122)	0.005
Serum albumin (g/L)	35.0 (3.2)	25.8 (4.1)	< 0.001
Uric Acid (μmol/L)	290 (32)	546 (324)	0.042
Creatinine (μmol/L)	44 (8)	59 (44)	0.002
Proteinuria	1/23 (4.3%)	9/14 (64.3%)	< 0.001
**Outcomes**			
Termination of pregnancy	23/23 (100%)	12/14 (85.7%)	–
Gestational age at delivery (wks)	25.4 (8.9)	29.1 (5.9)	0.034
Cesarean section	0/23 (0)	7/14 (50%)	< 0.001
Placental edema	15/23 (65.2%)	14/14 (100%)	0.015
Maternal morbidity	2/23 (8.7%)	12/14 (85.7%)	< 0.001
Postpartum hemorrhage	1/23 (4.3%)	7/14 (50%)	0.002
Placenta accreta	2/23 (8.7%)	5/14 (35.7%)	0.08
Other complications	0/23 (0)	10/14 (71.4%)	< 0.001
Blood products transfusion	1/23 (4.3%)	9/14 (64.3%)	< 0.001
Intensive care unit admission	0/23 (0)	6/14 (42.9%)	0.001
Length of hospital stay (days)	5 (3)	11 (5)	< 0.001
Cost (dollars)	582 (483)	3597 (4023)	< 0.001

Values are median (interquartile range) or number (percentage)

### Risk factors for MS

MS is characterized as “triple edema”, and fetal and/or placental edema are precursors of maternal edematic manifestations. Therefore, the fetal and placental parameters associated with the edematous manifestations were chosen as risk factors of MS among cases with fetal hydrops. Binary logistic regression analysis showed that at time of fetal hydrops onset ≥24 weeks (odd ration [OR] 15.83, 95% confidence interval [CI] 1.56–160.10) and placental thickening (OR 8.63, 95% CI 1.29–57.72) were risk factors related to the MS (Table 2).

**Table 2:** Logistic regression analysis of risk factors for mirror syndrome

Variables	OR (95% CI)	*P* value
Time of fetal hydrops onset ≥24 weeks	15.83 (1.56–160.10)	0.019
Placental thickening	8.63 (1.29–57.72)	0.026

*Abbreviation*: *OR* Odds ratio, *CI* Confidence interval

## Discussion

Although MS is a rare obstetric disease, the present case-control study found that it was relatively prevalent among fetal hydrops cases with an incidence of 37.8% (14/37) in the late second and third trimesters. The most common etiology of hydropic fetuses in southern China was alpha thalassemia major, and the fetal prognosis was very poor in both MS and non-MS groups. However, in the MS group, maternal morbidity increased with higher rates of postpartum hemorrhage, blood products transfusions, and ICU admissions; the key maternal features of MS included more weight gain, hemodilution and hypertension. In addition, our findings indicated that the time of onset of fetal hydrops at ≥24 weeks and prenatal placental thickening were risk factors for MS.

Since maternal edema is related to fetal hydrops and placental edema, any of the conditions associated with fetal hydrops or placental edema may cause MS. In southern China, alpha thalassemia major, which accounts for 28.4–55.1% of non-immune hydrops fetalis [10, 11], is also the common cause of MS. In our study, nearly half (17/37, 45.9%) of hydropic fetuses in the second and third trimesters were caused by alpha thalassemia major, and seven cases (7/17, 41.2%) developed MS. The high proportion of alpha thalassemia major in our study was inconsistent with frequently reported Rh-isoimmunization and viral infections in Western countries, which could be explained by differences in regions and populations. The other known etiologies included TTTS, fetal congenital structural anomalies, and placental chorioangioma, which were also common in previous studies [2, 3].

Although the maternal features of MS varied widely, all patients in the MS group manifested consistent symptoms of edema and hemodilution, characterized by lower extremity pitting edema, ascites, pleural effusion, anemia and hypoalbuminemia. Four patients presented with hypertension and proteinuria, and two of them developed HELLP syndrome before giving birth. Some studies have found that the changes in multiple placental factors associated with MS have the same pattern as preeclampsia [5–8]. This overlap between MS and preeclampsia can easily lead to misdiagnosis. However, consistent with previously reported cases of MS with maternal hemodilution [2, 3, 12, 13], our laboratory findings showed that the levels of hemoglobin, hematocrit, platelets and serum albumin were significantly lower in the MS group than in the non-MS group. Regardless of the fetal and placental causes, the hemodilution observed in cases of MS compared with typical hemoconcentration of preeclampsia may be considered in the differential diagnosis of these two diseases.

The prognosis of hydropic fetuses is generally poor and depends on the etiology and antenatal therapy [2, 3, 13]. In our study, the causes of hydrops were identified prenatally in 67.6% (25/37) of patients, and the most common etiology was alpha thalassemia major (17/37). All the parents in the non-MS group and 85.7% (12/14) in the MS group opted to terminate their pregnancies due to unsatisfactory guarantee for good fetal prognosis or worsening maternal conditions. Similar to previous reports [2, 3], no maternal mortality occurred in the MS group. However, maternal morbidity was as high as 85.7%, which was significantly higher than 8.7% in the non-MS group. Postpartum hemorrhage occurred in half of the patients in the MS group and the potential causes included placenta accreta, placentomegaly and maternal hemodilution, which are also features of MS. Moreover, nearly two-thirds of the patients in our study needed blood product transfusions and nearly half underwent ICU admission due to acute maternal heart failure, pulmonary edema, or DIC. However, the median time for resolution of maternal manifestations was 7.5 days (range: 2–10 days) after delivery, which was similar to the 5.5 and 8.9 days reported in other literatures [2, 3]. These adverse maternal outcomes and the subsequent resolution following delivery highlight the importance of early diagnosis and timely treatment of MS.

In this study, 37.8% (14/37) of fetal hydrops cases progressed to MS, implying that MS was relatively common among fetal hydrops cases, even though the actual incidence of MS at our hospital was as low as 0.024%. Thus, investigating the potential risk factors of MS is important for the early detection and close surveillance of high-risk pregnant women. However, to the best of our knowledge, there are no published studies on the risk factors for MS due to a paucity of cases or lack of awareness of this problem. MS mainly occurs during the late second and third trimesters [2, 3]. Among our MS cases, the median gestational age of fetal hydrops onset was 27.8 weeks, which was close to a previous report of 26.4 weeks [3] and significantly later than the median of 23 weeks in the non-MS group. Multivariate analysis showed that the time of fetal hydrops onset at ≥24 gestational weeks was an independent risk factor for MS; therefore, more attention should be paid to this high-risk population. Although the pathogenesis of MS is still unclear, some studies have suggested that placental abnormalities may be the inciting etiology [14, 15]. Our results showed that placental thickening was also a risk factor for MS. This finding highlights the importance of evaluating the placenta in hydropic fetalis cases.

Some limitations of our study should be discussed. First, despite MS being a rare obstetric condition, this retrospective study from a single center with a small sample may lead to selection bias. Second, nearly all patients in this study opted to terminate their pregnancies following diagnosis of fetal hydrops, and the actual rate of fetal survival and the efficacy of intrauterine interventions could not be assessed. Lastly, clinical data did not consider any pathophysiologic markers, and the mechanism of MS could not be discussed herein.

## Conclusion

MS is a common obstetric disease among fetal hydrops cases in late second and third trimesters. The main cause of fetal hydrops and also MS is alpha thalassemia major in southern China. With diverse maternal manifestations, the key features include more weight gain, hemodilution, and hypertension. The high maternal morbidity is mainly attributed to postpartum hemorrhage. In addition, this study demonstrated that the time of onset of fetal hydrops at ≥24 weeks and prenatal placental thickening are both risk factors for MS; thus, more attention should be paid to high-risk populations that have these characteristics. Additional prospective studies with larger study populations of fetal hydrops and MS cases are needed to further assess these risk factors.

## Supplementary Information

**Additional file 1.**

## Data Availability

The datasets used and/or analysed during the current study are available from the corresponding authors on reasonable request.

## Abbreviations

MS: Mirror syndrome
ICU: Intensive care unit
TTTS: Twin-to-twin transfusion syndrome
HELLP: Hemolysis, elevated liver enzymes, low platelet count syndrome
DIC: Disseminated intravascular coagulation
SD: Standard deviations
OR: Odd ration
CI: Confidence interval
