# Supplementary material for: Clinical characteristics and risk factors of mirror syndrome: a retrospective case-control study
Source: BMC Pregnancy Childbirth. 2021 Sep 28;21:660. doi: 10.1186/s12884-021-04143-3 (PMC8480018; doi:10.1186/s12884-021-04143-3)
Supplement: Supplementary file 1 — Additional file 1. [file 12884_2021_4143_MOESM1_ESM.docx]

**Supplementary table 1** Clinical characteristics and outcomes of 14 pregnant women with mirror syndrome

| Patient No. | Etiology | Time of fetal hydrops onset (wks) | Prenatal sonographic findings | placental thickness (mm) | Time of diagnosis of mirror synrome (wks) | Maternal manifestation | Gestational age at delivery (wks) | Mode of delivery | Maternal complication | Fetal outcomes | Placental examination |
| --- | --- | --- | --- | --- | --- | --- | --- | --- | --- | --- | --- |
| 1 | Fetal anemia (Normal thalassemia testing and karyotyping) | 29 | Generalized subcutaneous edema, pleural effusion, polyhydramnios | 40 | 28 | Lower limbs edema, ascites, hypoalbuminemia, anemia, proteinuria | 29.3 | Cesarean section | Acute pyelonephritis, renal dysfunction | Female, Wt 2500g, Ht 30cm, generalized subcutaneous edema, Apgar score: 1-0-0 | Wt 750g, thickening and edema. Villi focal fibrinoid degeneration, chorioamnionitis |
| 2 | Alpha thalassemia major | 27 | Generalized subcutaneous edema, pericardial effusion, pleural effusion, oligohydramnios | 89 | 34 | Lower limbs edema, hypoalbuminemia, anemia, proteinuria | 35.3 | Induced vaginal delivery | Placental accreta, postpartum hemorrhage | Male, Wt 2400g, Ht 44cm, stillbirth, generalized subcutaneous edema | Wt 1930g, thickening and edema. Nucleated red blood cells in the villi, interstitial edema, mild nodular hyperplasia of somatic trophoblasts |
| 3 | Unknown (Normal karyotyping) | 25.7 | Subcutaneous edema of fetal right lower limb, cardiomegaly, pericardial effusion, polyhydramnios | 61 | 25.3 | Lower limbs edema, anemia | 25.9 | Cesarean section | Placental abruption, DIC, postpartum hemorrhage | Male, Wt 960g, Ht 35cm, stillbirth, subcutaneous edema of right lower limb | Wt 580g, thickening and edema. Nucleated red blood cells in the villi blood vessels, interstitial edema |
| 4 | Alpha thalassemia major | 32 | Generalized subcutaneous edema, cardiomegaly, ascites, polyhydramnios | 72 | 26 | Lower limbs and abdominal wall edema, hypoalbuminemia, anemia, proteinuria | 34 | Induced vaginal delivery | Placental accreta, gestational diabetes | Male, Wt 2575g, Ht 44cm, stillbirth, generalized subcutaneous edema, hypospadias | Wt 1250g, thickening and edema. Nucleated red blood cells in the villi vessels, interstitial edema |
| 5 | Alpha thalassemia major | 26.7 | Ascites, pleural effusion, oligohydramnios | 65 | 25.3 | Hypertension, pleural effusion, ascites, hypoalbuminemia, anemia, proteinuria | 27.7 | Cesarean section | Placenta previa, acute left heart failure, postpartum hemorrhage | Male, Wt 1525g, Ht 40cm, intrauterine fetal death, abdominal distension | Wt 1390g, thickening and edema. Villi fibrinous degeneration, with nucleated red blood cells in the blood vessels of the villi |
| 6 | TTTS stage IV | 25.7 | One twin has generalized subcutaneous edema, cardiomegaly, polyhydramnios | 34 | 25.9 | Pleural effusion, pericardial effusion, hypoalbuminemia, anemia | 25.9 | Cesarean section | Acute left heart failure, pulmonary edema | Two females, one twin with subcutaneous edema: Wt 980g, Ht 36cm, Apgar score: 8-9-9; co-twin: Wt 680g, Ht 29cm, Apgar score:8-9-9 | Wt 590g. The placental area of bigger twin is 3 times that smaller co-twin. Partial villous fibrinous degeneration with small focal calcification, mild nodular hyperplasia of syncytiotrophoblasts |
| 7 | Unknown | 30.3 | Generalized subcutaneous edema, cardiomegaly, pleural effusion | 63 | 30 | Hypertension, HELLP syndrome, pleural effusion, ascites, hypoalbuminemia, anemia, proteinuria | 31.6 | Spontaneous vaginal delivery | Renal dysfunction, pneumonia, placental accreta, postpartum hemorrhage | Male, Wt 2300g, Ht 39cm, stillbirth, generalized subcutaneous edema, short lower limbs | Wt 1000g, thickening and edema. Focal villous fibrinoid degeneration, mild nodular hyperplasia of syncytiotrophoblasts |
| 8 | Ebstein anomaly (Normal karyotyping and SNP-array) | 27.4 | Generalized subcutaneous edema, cardiomegaly with the tricuspid valve down and enlarged foramen ovale, pericardial effusion, pleural effusion, ascites | 46 | 26.4 | Lower limbs edema, hypoproteinemia, anemia, proteinuria | 28.3 | Cesarean section | Acute left heart failure, pulmonary edema, gestational diabetes | Male, Wt 2100g, Ht 41cm, stillbirth, generalized subcutaneous edema, abdominal distension | Wt 820g, thickening and edema. Some villous cellulosic degeneration, mild nodular hyperplasia of syncytiotrophoblasts |
| 9 | Alpha thalassemia major | 28.1 | Generalized subcutaneous edema, pleural effusion | 60 | 28.1 | Hypertension, HELLP syndrome, pleural effusion, ascites, hypoproteinemia, anemia, proteinuria | 29 | Cesarean section | Renal dysfunction, postpartum hemorrhage, pneumonia | Male, Wt 1410g, Ht 30cm, stillbirth, generalized subcutaneous edema | Wt 1100g, thickening and edema. Some villous fibrinous degeneration, poor development of interstitial blood vessels, many nucleated red blood cells in the villous vessels |
| 10 | Alpha thalassemia major | 22.6 | Generalized subcutaneous edema, cardiomegaly, pleural effusion, ascites, oligohydramnios | 50 | 22 | Lower limbs edema, hypoproteinemia, anemia | 22.6 | Cesarean section | Liver dysfuction | Female, Wt 2000g, Ht 43cm, stillbirth, generalized subcutaneous edema | Wt 1100g, thickening and edema. Local fibrinoid degeneration |
| 11 | Alpha thalassemia major | 33.6 | Cardiomegaly, pericardial effusion, ascites | 62 | 33 | Lower limbs edema, hypertension, ascites, hypoproteinemia, anemia | 34.6 | Induced vaginal delivery | Placental accreta, postpartum hemorrhage | Female, Wt 2400g, Ht 42cm, stillbirth, generalized subcutaneous edema, abdominal distension | Wt 1160g, thickening and edema. Local villi degeneration and necrosis, nucleated red blood cells in the villi vessels |
| 12 | Alpha thalassemia major | 29.1 | Generalized subcutaneous edema, cardiomegaly, pericardial effusion, pleural effusion, ascites | 73 | 28.1 | Lower limbs edema, hypertension, pleural effusion, ascites, hypoalbuminemia, anemia, proteinuria | 31.7 | Spontaneous vaginal delivery | Placental accreta, postpartum hemorrhage | Female, Wt 2230g, Ht 40cm, stillbirth, generalized subcutaneous edema, abdominal distension, short lower limbs | Wt 1200g, thickening and edema. Interstitial edema, villi vessels markedly reduced, nucleated red blood cells in the vessels |
| 13 | Unknown (Normal karyotyping and SNP-array) | 32.4 | Generalized subcutaneous edema, pleural effusion, ascites, polyhydramnios | 26 | 31.9 | Lower limbs edema, pericardial effusion, hypoproteinemia, anemia | 32.9 | Induced vaginal delivery | None | Female, Wt 3150g, Ht 43cm, stillbirth, generalized subcutaneous edema, abdominal distension | Wt 850g, thickening and edema. Villi fibrinous degeneration, interstitial edema |
| 14 | Cardiac (Normal karyotyping and SNP-array) | 26.1 | Generalized subcutaneous edema, cardiomegaly, pleural effusion, ascites, polyhydramnios | 43 | 25 | Lower limbs edema, anemia | 27.7 | Induced vaginal delivery | None | Female, Wt 1210g, Ht 30cm, stillbirth, generalized subcutaneous edema, abdominal distension | Wt 640g, thickening and edema. Villi fibrinous degeneration, mild nodular hyperplasia of syncytiotrophoblasts, interstitial edema |
|  | | | | | | | | | | | |
